# Characterization of the Floral Transcriptome of Moso Bamboo (*Phyllostachys edulis*) at Different Flowering Developmental Stages by Transcriptome Sequencing and RNA-Seq Analysis

**DOI:** 10.1371/journal.pone.0098910

**Published:** 2014-06-10

**Authors:** Jian Gao, Ying Zhang, Chunling Zhang, Feiyan Qi, Xueping Li, Shaohua Mu, Zhenhua Peng

**Affiliations:** International Center for Bamboo and Rattan, Key Laboratory of Bamboo and Rattan Science and Technology, State Forestry Administration, Beijing, People's Republic of China; University of Heidelberg, Germany

## Abstract

**Background:**

As an arborescent and perennial plant, Moso bamboo (*Phyllostachys edulis* (Carrière) J. Houzeau, synonym *Phyllostachys heterocycla* Carrière) is characterized by its infrequent sexual reproduction with flowering intervals ranging from several to more than a hundred years. However, little bamboo genomic research has been conducted on this due to a variety of reasons. Here, for the first time, we investigated the transcriptome of developing flowers in Moso bamboo by using high-throughput Illumina GAII sequencing and mapping short reads to the Moso bamboo genome and reference genes. We performed RNA-seq analysis on four important stages of flower development, and obtained extensive gene and transcript abundance data for the floral transcriptome of this key bamboo species.

**Results:**

We constructed a cDNA library using equal amounts of RNA from Moso bamboo leaf samples from non-flowering plants (CK) and mixed flower samples (F) of four flower development stages. We generated more than 67 million reads from each of the CK and F samples. About 70% of the reads could be uniquely mapped to the Moso bamboo genome and the reference genes. Genes detected at each stage were categorized to putative functional categories based on their expression patterns. The analysis of RNA-seq data of bamboo flowering tissues at different developmental stages reveals key gene expression properties during the flower development of bamboo.

**Conclusion:**

We showed that a combination of transcriptome sequencing and RNA-seq analysis was a powerful approach to identifying candidate genes related to floral transition and flower development in bamboo species. The results give a better insight into the mechanisms of Moso bamboo flowering and ageing. This transcriptomic data also provides an important gene resource for improving breeding for Moso bamboo.

## Introduction

Transcriptome sequencing is a convenient way of rapidly obtaining information on the expressed fraction of a genome [1]. Next-generation sequencing (NGS) technology, such as the Illumina Solexa, Roche 454 and ABI SOLiD platforms, has emerged as a cost-effective approach for high-throughput sequencing. It has revolutionized biological research by rapidly providing genomic and transcriptomic data [2]–[4]. RNA-Seq refers to the whole-transcriptome shotgun sequencing of fragmented mRNA or cDNA, resulting in overlapping short reads covering the entire transcriptome [5], [6]. RNA-Seq analysis is powerful, enabling large-scale functional assignments of genes via the assembly of large sequenced transcriptome library. With this technique, quantitative analysis of gene expression can be easily performed without potential bias, allowing a more sensitive and accurate profiling of the transcriptome [7], [8]. Despite its obvious potentials, this combined approach has not yet been applied to Moso bamboo (*Phyllostachys edulis* (Carrière) J. Houzeau, synonym *Phyllostachys heterocycla* Carrière) [9] research.

Bamboo is one of the most important non-timber forest products in the world. About 2.5 billion people depend on bamboo, ranging from food to raw materials for construction and manufacture [10]–[12]. Bamboos (Bambusoideae) belong to the monophyletic BEP clade (Bambusoideae, Ehrhartoideae, Pooideae) in the grass family (Poaceae), and consist of woody and herbaceous species. Woody bamboos are arborescent, perennial plants characterized by their woody stems and a prolonged vegetative phase lasting decades or even longer before flowering [13]. In addition, they often flower synchronously and die collectively after flowering. Two types of bamboo are usually recognized – sympodial bamboos, which form clumps of culms, and monpodial bamboos which form groves. Under normal circumstances, flowering of monopodial bamboo rarely occurs. However, collective death in some bamboo species has occasionally occurred after flowering over a large area. The reasons for this remain unclear, but it is a devastating blow to bamboo resource base. Therefore, it is important to determine the specific pathways and genes involved in bamboo flowering and flower development.

Several putative flowering-related genes have been identified from certain bamboo species [14]–[18], and environmental and chemical manipulations have been found to induce bamboo flowering *in vitro* [19]. Moreover, Zhang et al. studied the transcriptomes of developing flowers in *Dendrocalamus latiflorus* using Illumina sequencing [20].

As a large woody bamboo with high environmental, economic and cultural value in Asia [21], Moso bamboo is a perennial plant characterized by its infrequent sexual reproduction with flowering intervals ranging from several to more than a hundred years. Flowering depends on many factors, including environment, nutrition, climate and the plants' physiological status. Several hypotheses have been proposed for the flowering mechanisms [22], [23]. However, there have been few molecular studies on flowering of Moso for various reasons including the difficulty of collecting tissue samples, lack of genomic knowledge, infrequent sexual reproduction and long periods of time between flowering. With the announcement of the genome sequence of Moso bamboo [21], it is now possible to identify and determine the molecular regulation mechanisms of all functional elements in Moso bamboo. The transcriptome represents a comprehensive set of transcribed regions throughout the genome. Therefore, understanding the transcriptome dynamics is essential for revealing functional elements of the genome and interpreting phenotypic variation produced by combinations of genotypic and environmental factors.

In the present study, we aimed to investigate the transcriptome of developing flowers in Moso bamboo through high-throughput Illumina GAII sequencing and mapping short reads to the Moso bamboo genome and reference genes. Here, compared with the previous study reference [as 21], for the first time we collected from the same plant both leaves in the vegetative stage and flowering spikelet samples in different floral developmental stages. As transcriptome results are affected by the impact of external environmental conditions and growth differences among plants, transcriptome results from the same plant are more accurate to elucidate the molecular mechanism of regulation of flower development of bamboo.

Transcripts from four flower growth phases of the same plant were isolated, quantified and sequenced. The transcriptome sequences were then blasted against public databases. Subsequently, the annotated genes were clustered into putative functional categories using the Gene Ontology (GO) framework and grouped into pathways using the Kyoto Encyclopedia of Genes and Genomes (KEGG). Finally, we assigned genes putative homologs in model species, including *Arabidopsis thaliana*, *Oryza sativa*, *Brachypodium distachyon* and other relatives in the grass family to determine whether a hierarchy of gene regulation may persist between these species. Taken together, we, for the first time, comprehensively characterized the molecular basis of the physiological processes during the flower development of Moso bamboo using large-scale high-throughput sequencing, and our results may lay a foundation for further gene expression and functional genomic studies in bamboos.

## Results and Discussion

### Sequence data quality control and filtering

In order to get an overview of the Moso bamboo transcriptome, we generated cDNA libraries from leaves of non-flowering plants (abbreviated as CK) and equally mixed RNA extracted from panicles at four different flowering developmental stages (abbreviated as F) using paired-end sequencing by the Illumina platform. In total, we acquired more than 67 million reads from each of the CK and F samples. Through mapping all these short reads to the reference genome of Moso bamboo, we found that about 70% of the reads could be uniquely mapped to the genome. Gene coverage was determined as the percentage of a gene covered by reads. 15,497 and 167,767 reads were detected from each of the F and CK samples respectively, with gene coverage from 90% to 100% (Figure S1).

### The reads mapping to the reference genome dataset

To identify the genes corresponding to these clean reads in each library, the clean reads were mapped to the reference genes expressed in the Moso bamboo genome. Mapping results showed that 39,026,185 and 39,386,902 reads from each library were perfectly matched to the reference genome while about 22,824,394 and 23,064,627 reads were perfectly matched to the reference genes (Table 1). The reads of unique matched to genome were 47,847,557 and 47,709,687, and those matched to reference genes were 30,640,507 and 30,868,269 in the two libraries. Altogether, there were 52,489,347 and 53,035,796 reads matched to the reference genome, and 31,843,985 and 32,346,195 reads matched to the reference genes. However, as a result of the significant sequencing depth of Illumina technology and incomplete annotation of the Moso bamboo genome, 15,815,789 and 14,582,070 unmatched reads to the reference genome and 36,461,151 and 35,271,671 unmatched reads to the reference genes in each library were observed. The alignment statistics results of these reads from library CK and F are also shown in Table 1.

**Table 1:** Alignment statistics of sample CK and F.

Tissue	F	CK
Total Reads	68305136	67617866
Total BasePairs	6147462240	6085607940
Map to Genome		
**Total mapped reads**	52489347	53035796
**Perfect match**	39026185	39386902
**< = 5bp mismatch**	13463162	13648894
**Unique match**	47847557	47709687
**Multi-position match**	4641790	5326109
**Total unmapped reads**	15815789	14582070
Map to Gene		
**Total mapped reads**	31843985	32346195
**Perfect match**	22824394	23064627
**< = 5bp mismatch**	9019591	9281568
**Unique match**	30640507	30868269
**Multi-position match**	1203478	1477926
**Total unmapped reads**	36461151	35271671

### GO annotation and KEGG pathway mapping

Based on sequence homology, a total of 18,309 genes were categorized into the three main GO categories (biological process, cellular component and molecular function). The GO analysis showed that the identified genes were involved in flower development. In the GO classification, “pollen wall” (6,420, 94.3%), “exine” (6,420, 94.3%) and “sexine” (5,027, 73.8%) terms were dominant, respectively. We also noticed that genes of “spindle microtubule” (5,590, 82.1%), “ornithine transport” (4,123, 71.5%), “ketone biosynthetic process” (4,119, 71.4%), “actin filament severing” (3,087, 53.5%) and “regulation of meiotic cell cycle” (3,080, 53.4%) were well represented. Moreover, additional table S1 shows that 1,794 genes were annotated as “movement in host environment” category, suggesting a potential connection between environment and flowering. Functional classification and pathway assignment were performed by the KEGG [24]. A total of 23,522 genes were mapped into 302 KEGG pathways, representing compound biosynthesis, utilization, metabolism and degradation. The largest category was metabolic pathways which included 7,316 genes. Additional table S2 shows that transcripts related to genetic information processing and environmental information processing were the most abundant such as RNA transport (Ko03013, 3,921 genes, 16.67%), mRNA surveillance pathway (Ko03015, 3,301 genes, 14.03%), microbial metabolism in diverse environments (Ko01120, 1,621 genes, 6.89%), plant-pathogen interaction (Ko04626, 1,582 genes, 6.73%) and plant hormone signal transduction (Ko04075, 1,293 genes, 5.5%).

### Putative Moso bamboo floral development transcription factors

One unique characteristic of Moso bamboo is the switch to flowering after a very long period of vegetative growth and the undertermined flowering time. In order to compare the gene expression patterns between flowering and vegetative tissues, we collected panicles and vegetative leaves from non-flowering Moso bamboo plants for Illumina sequencing analysis. More than 714 floral development-related genes were up-regulated in the panicle tissues (with at least a 2-fold difference in the expression level in panicles compared with the level in leaves), including 476 flowering genes in the category of transcription factor genes (Table 2, Table S3 and Table S4).

**Table 2:** The genes related to transcription factor families.

Transcription Factor Families	Gene Numbers
MADS-Box gene (MADS)	38
heat shock transcription factor 20 (HSP20)	28
basic helix-loop-helix factor (bHLH)	34
basic leucine zipper (bZIP)	6
NAM-ATAF-CUC family (NAC)	61
heat shock transcription factor 70 (HSP70)	1
WRKY transcription factor (WRKY)	110
MYB transcription factor (MYB)	143
DNA-binding with-one finger (Dof)	15
AP2/EREBP transcription factor gene (AP2/EREBP)	32
Heat Shock Transcription Factor (HSF)	28
high-mobility group box (HMG)	4

Transcriptional regulation is mediated through the interplay between transcription factors and specific-regulatory regions of the genome, leading to gene activation or other changes [25]. Therefore, some transcription factors may be considered as master regulators, since their actions initiate a branching series of downstream effects, including the activation of other transcription factors, ultimately resulting in broad changes in the organism (as [19]). Our study focused on these key regulatory genes in the flower development of Moso bamboo. A total of 476 putative transcription factor families were identified in panicle tissues of Moso bamboo, including WRKY (110), MADS-Box (38), bHLH (34), Dof (15), NAC (61), HSF (4), HMG (4), bZIP (6), HSP20 (28), HSP70 (1), MYB (143) and AP2/EREBP (32).

MADS-box transcription factors contain a DNA binding domain conserved among eukaryotes. Many MADS family members have been intensely studied in model plants, with the floral-organ identity and development proteins falling into the MIKC clade [26]–[28], such as homologs of MADS1, MADS2, MADS5, MADS7, MADS12, MADS14, MADS15 and MADS56 in *Brachypodium distachyon*, *Oryza sativa Japonica* Group and *Fargesia nitida*. MADS family members have been shown to orchestrate floral organ specification and development [20], [26]. Loss-of-function mutants of MADS-box genes have caused changes in organ identity. In this study, a total of 38 MADS-Box transcription factors were identified in Moso bamboo flowers, which are homologs of MADS1, MADS2, MADS4, MADS7, MADS11, MADS14, MADS15, MADS17, MADS27, MADS31 and MADS58 from *Zea mays*, *Fargesia nitida*, *Oryza sativa Indica* Group and *Brachypodium distachyon*. Genes *OsMADS14*, *OsMADS15* (rice FUL1-like gene) is expressed only in inflorescence and developing caryopses [29]. *OsMADS18* (rice FUL3-like gene) causes an early flowering phenotype and early initiation of axilliary shoot meristems. It indicates that *OsMADS18* acts as a promoter in the differentiation activity of the vegetative shoot [30]. Dreni et al indicated that *OsMADS3* and *OsMADS58* might have a redundantly function in specifying floral organ identity, including the carpel [31].

Dof (DNA binding with one zinc finger) transcription factors are a group of plant-specific transcription factors containing a single Cys2/Cys2-type zinc-finger-like Dof motif [32], [33]. There are 37 Dof transcription factors in the Arabidopsis genome, and all of them have a highly conserved DNA-binding domain and divergent sequences beyond this domain [33]–[36]. They are involved in diverse functions, such as seed dormancy [37], carbon metabolism [38], phytochrome signaling pathway [39], phenylpropanoid metabolism [40] and flowering response [41], [42]. *JcDof3* is a circadian clock regulated gene, and it might be involved in the regulation of flowering time in *Jatropha curcas* [43]. Li et al. suggested that *OsDof12* over-expression induces an early flowering under long-day (LD) conditions, the transcription levels of *Hd3a* and *OsMADS14* were up-regulated [44]. We detected 28 putative Dof transcription factors in panicles, which are homologs of Dof3, Dof4, Dof5, Dof12 and CDF (Cycling DOF Factor) family, revealing similar roles of Dof 3 and Dof 12 genes during the flower development of Moso bamboo. We also identified *Hd3a*, a master floral developmental regulator in rice (with apparent paralogous counterparts in *Arabidopsis* called *FT*). Peng et al. (2013) suggested that drought or other environmental stresses are functional in regulating MADS14s in the flowering stage of bamboo (as [21]). Taken together, an active pathway of Dof-Hd3a-MADS-flowering may play an important role during the Moso bamboo flowering.

WRKY transcription factors are associated with senescence [45], [46] and stress responses [47]. Members of this family have been identified as important downstream components of MAPK signaling pathways (as [19]). The involvement of WRKY factors in plant defense is also well documented: some of them have been shown to confer disease resistance [48], [49], triggering the expression of defense-related genes in systemic acquired resistance [50]–[53]. They may be induced by signaling hormones, such as salicylic acid [54], [55], jasmonic acid [56] and gibberellic acid [57]. In the present study, putative homologs of WRKY transcription factors exhibited a similar over-representation, with 110 genes showing high abundance in panicles of Moso bamboo.

The basic helix-loop-helix family (bHLH) consists of genes regulating various processes of flower development, such as controlling the development of carpel margins, as well as the morphogenesis of sepals, petals, stamens and anthers in *Arabidopsis thaliana* and *Eschscholzia californica* [58]. In our study, 34 bHLH-like genes were highly expressed in panicle tissues.

MYB transcription factors contain DNA binding domains, and some have been identified as floral developmental regulators [59]. In Arabidopsis, MYB21, MYB24 and MYB57 are crucial factors for the development of stamen filament. It has been reported that *R2R3-MYB* genes regulate various metabolic pathways, contributing to the control of flavonoid biosynthesis, tryptophan biosynthesis and participating in phenylpropanoid metabolism [60], [61]. In plants, basic leucine zipper motif (bZIP) transcription factors regulate a series of processes, including pathogen defense, light and stress signaling, seed maturation and flower development. We found that these upstream regulatory stress-responsive genes were highly expressed during flowering, suggesting a potential connection between drought, pathogen or senescence and bamboo flowering.

### Detection of putative genes related to flowering time control and flower development

Previous studies of model plant species showed that the timing of floral induction is controlled by sophisticated regulatory networks that monitor changes in the environment, including autonomous pathway, photoperiod and circadian clock pathway, gibberellins pathway, ambient temperature pathway and age pathway [62], [63]. The unusual and infrequent nature of bamboo flowering has attracted the curiosity of scientists and laypeople for centuries [64]. However, little research has been conducted about the molecular mechanism of bamboo flowering due to the difficulty of collecting flowering samples. Through the comparison of Moso bamboo genes found in this study with the NCBI and Uniprot databases, we identified at least 238 genes as homologs of known flowering-related genes from other plants (Table S3). These genes were compared with flowering genes from *Oryza sativa*, *Hordeum vulgare*, *Brachypodium distachyon*, *Sorghum bicolor* and *Arabidopsis thaliana*. However, we found that the genes employed in typical flowering promotion pathways (such as those autonomous pathway, ambient-temperature) and floral pathway integrator (FPI) genes (as [63], [65]) were not highly expressed in these floral tissues in bamboo. Only late elongated hypocotyl (*LHY*), phytochrome-interacting facor 3 (*PIF3*), crytochrome-1 (*CRY1*), GIGANTEA (*GI*) and CONSTANS (*CO*) genes were detected in Moso bamboo for the photoperiod and circadian clock pathway. We did not detect any genes homologous to components of the circadian clock and photoperiod pathway, such as early flowering 3 (*ELF3*), early flowering 4 (*ELF4*), circadian clock associated 1 (*CCA1*), timing of CAB1 (*TOC1*), pseudo response regulator (*PRR5*, *PRR7* and *PRR9*) and phtyochrome (*PHYA*, *PHYB*) [66]. The *CO* gene plays a key role in integrating light and temporal information, and it is essential for determining the flowering time [67]. The CO protein consists of two zinc fingers, including a N-terminal B-domain mediating protein-protein interactions and a C-terminal CCT domain for nuclear localization [68]. In Arabidopsis, CO, as a transcription factor, promotes flowering by inducing the expression of flowering locus T (FT) [69]. Rice orthologues of the Arabidopsis genes *CO* and *FT* have been identified as *Hd1* and *Hd3a*, respectively; and *Hd1* controls rice flowering by regulating *Hd3a* (as [57]). In the present study, we found that 41 putative CO transcription factors were involved in flower development. However, most of them were down-regulated in floral tissues of Moso bamboo. The upstream floral repressor factors, such as LHY and PIF3, were highly expressed, whereas the FPI genes (*GI* and *CRY1*) were down-regulated, which might result in the low expression of *CO* genes. Low expression of *CO* genes suggested that the flower initiation was more independent of these traditional promotion pathways in bamboo flowering.

Genes involved in gibberellins pathway have high expression, which is known as the promotion pathways in sophisticated regulatory networks. GA regulates diverse developmental processes during plant growth, ranging from seed germination, leaf expansion, and stem elongation to floral initiation [70]. Putative homologs of GA-signaling pathway genes were highly expressed, including the gibberellin response modulator (GAMYB) [71] and gibberellin receptors (*GID1*, *GID2*) [72]. Gibberellin triggers degradation of the DELLAs (such as *GAI*, *RGA*, and *RGL78-80*) and up-regulates *MYB21*, *MYB24* and *MYB57*, which are essential for stamen filament growth (as [59]). High expression of these genes indicated the connection between GA and bamboo flowering.

Unlike the flowering pathway genes, over 252 genes involved in the response to drought stress or to other correlative stresses (such as oxidative stress) were highly expressed. Some FMI-related genes and their upstream regulatory drought-responsive genes were highly expressed during flowering, such as *Dof3*, *Dof5*, *Dof7*, *Dof10*, *Dof12* and bZIP transcription factors. Putative homologs of FMI genes [73]–[75], such as *AP1* and *AP2*, *MADS1*, *MADS14* and *MADS15*, were also highly expressed. High expression of these above-mentioned genes indicated a potential connection between drought or other environmental stress and bamboo flowering.

### Changes in gene expression profiles during different flower developmental stages

**Figure 1:**
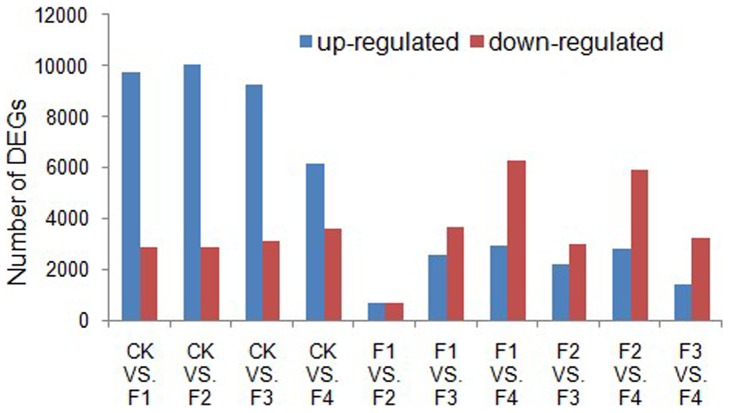
Changes in gene expression profile among the different flower developmental stages. The number of up-regulated and down-regulated genes between CK and F1; CK and F2; CK and F3; CK and F4; F1 and F2; F1 and F3; F1 and F4; F2 and F3; F2 and F4; F3 and F4 are summarized. DEGs: Differentially Expressed Genes.

DGEs (Differentially Expressed Genes) generates absolute rather than relative gene expression measurements and avoids many inherent limitations of microarrays (as [8]). DGEs were used to analyze the gene expression during four stages of flower development in Moso bamboo. Four cDNA libraries (F1, F2, F3 and F4) were sequenced, resulting in between 10 to 11 million high-quality reads. The read sequences were mapped to Moso bamboo reference transcriptome database. About 8,412,664 to 9,367,437 reads were mapped to a gene in the reference genome database, and up to 80% of the sequences in transcriptome could be unequivocally identified to the Moso bamboo genome. About 5,185,207 to 5,820,078 reads were mapped to a gene in the reference gene database, and up to 79% of the sequences in transcriptome could be perfect matched to the Moso bamboo genes (Table 3). Additional Figure S2 shows the quality assessment of reads, sequencing saturation analysis, randomness assessment and gene coverage. We identified differentially expressed genes between samples using a previously developed algorithm by Audic and Claverie [76]. As expected, the majority of gene expression changes occurred between the CK and F1/F2/F3/F4 samples, with more up-regulated genes observed (Figure 1 & Table S5, S6, S7, and S8). Moreover, about 1% (Table S9) of the up-regulated genes were found to be orphan sequences, which were not annotated to the Gene Ontology database (http://www. Geneontology.org/) with nr annotation, but the expression level of genes were significantly different, so we speculate that these genes may take part in other unique processes and pathways involving in the flower development of Moso bamboo.

**Table 3:** Statistics of RNA-seq sequencing.

Tissue	F1	F2	F3	F4
Total Reads	10424052	11127400	11046632	10828810
Total BasePairs	510778548	545242600	541284968	530611690
Map to Genome				
**Total mapped reads**	8768572	9367437	9101401	8412664
**Perfect match**	7184271	7662051	7467544	6787731
**< = 2bp mismatch**	1584301	1705422	1633857	1624933
**Unique match**	7458759	7942000	7759666	7199619
**Multi-position match**	1309813	1425437	1341735	1213045
**Total unmapped reads**	1655480	1759963	1945231	2416146
Map to Gene				
**Total mapped reads**	5626520	5820078	5334090	5185207
**Perfect match**	4452869	4604128	4226029	4028547
**< = 2bp mismatch**	1173651	1215950	1108061	1156660
**Unique match**	5030921	5208332	4809727	4680929
**Multi-position match**	595599	611746	524363	504278
**Total unmapped reads**	4797532	5307322	5712542	5643603

### Expression levels of putative genes involved in flower development

**Figure 2:**
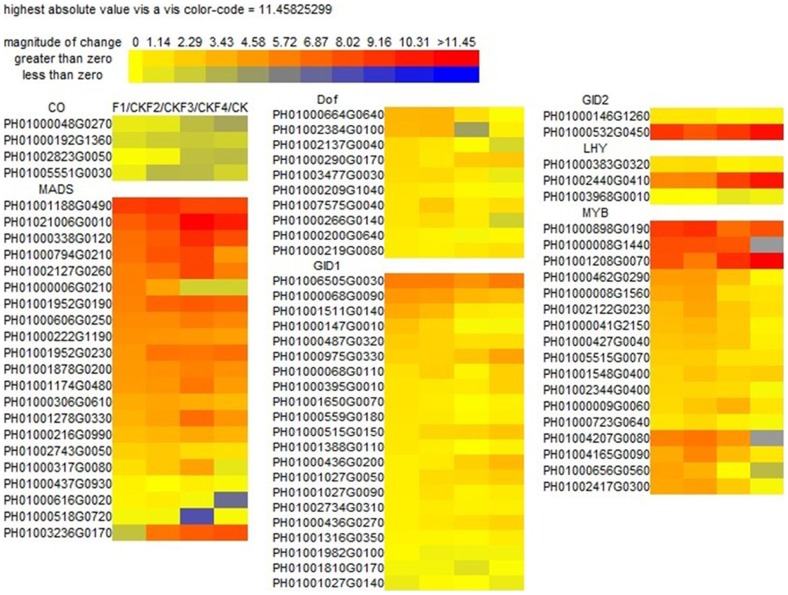
Clustering of genes involved in bamboo flowering expression profiles at four different flower developmental stages.

A total of 80 genes were annotated as flowering regulating pathway in Moso bamboo transcriptome (Table S10). To find whether these genes were putatively involved in flowering regulating pathway, expression profiles of these genes were compared with CK sample by DGEs (shown in Figure 2). From CK to F1 and from CK to F2, *MADS*, *Dof*, *GID1*, *GID2*, *LHY* and *MYB* genes were up-regulated. Some of these genes exhibited a decreased expression over time. The expression of *Dof* genes was significantly decreased in the anthesis and embryo formation stages, suggesting that *Dof* genes played a greater role in the early stages of flower development. Flowering inhibitors, such as *LHY*, *PIF3* genes, were up-regulated during flower developmental periods, which might result in the down-regulated expression of *CO* genes in floral tissues. Low expression of *CO* genes and high expression of stress-response genes and FMI genes suggested that the activation of FMI was more independent of the traditional flowering regulating pathway in Moso bamboo flowering.

### Gene validation and expression analysis

**Figure 3:**
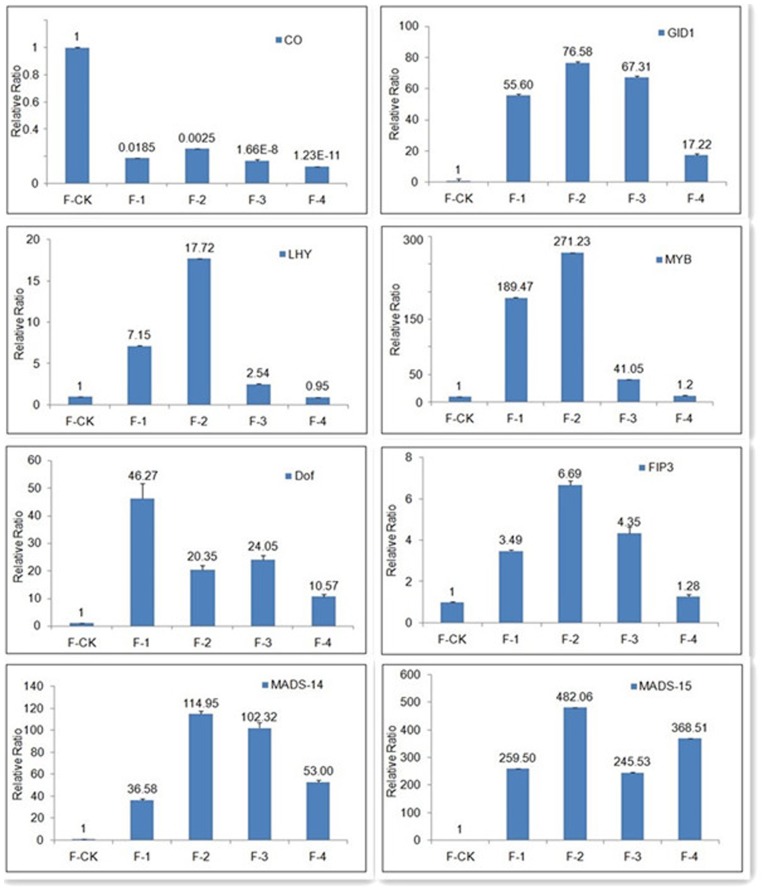
The expression profiles of 8 selected genes from flowering tissues in different flower developmental stages and leaves of non-flowering of Moso bamboo. The transcript levels were normalized to that of *TIP41* (tonoplast intrinsic protein 41), and the level of each gene in the control was set at 1.0. Error bars represent the SD for three independent experiments. *CO* (CONSTANS), *GID1* (gibberellin receptor GID1), *DOF* (Dof zinc finger protein 12), *MYB* (MYB transcription factor), *FIP3* (phytochrome- interacting factor 3), *MADS-14* (MADS-box gene-14), *MADS-15* (MADS-box gene-15), *LHY* (late elongated hypocotyl).

To confirm experimentally that the genes obtained from clean reads and computational analysis were indeed expressed, 8 putative genes related to flower development were chosen for RT-PCR and qRT-PCR analysis (Figure 3 and Table S11).

In the RT-PCR analysis, every selected gene was PCR positive with a single band at the calculated size (data not shown). According to the qRT-PCR results (Figure 3 and table 4), *CO* gene were down-regulated in floral tissues of Moso bamboo, whereas *LHY* and *PIF3*, which are the upstream floral repressor factors, up-regulated during the flower development. Drought-responsive genes such as *Dof* were highly expressed in floral tissues, especially in the early stage of flowering; *GID1* expression levels were similar to Dof in floral tissues. The expression levels of *MYB*, *MADS-14*, *MADS-15* in floral tissues were higher than those in non-flowering leaves. The results of qRT-PCR expression analysis matched the putative functions of Illumina analysis.

**Table 4:** The different expression of the 8 genes in different conditions of in different flowering developmental stages of *Phyllostachys edulis*.

Gene	F-CK		F-1		F-2		F-3		F-4	
	RPKM	log^2^ Ratio	RPKM	log^2^ Ratio	RPKM	log^2^ Ratio	RPKM	log^2^ Ratio	RPKM	log^2^ Ratio
*CO*(PH01000048G0270)	22.99	1	12.40	−0.89	11.58	−0.99	2.98	−2.95	1.539	−3.91
*GID1*(PH01000068G0090)	1.62	1	39.47	4.60	35.40	4.48	14.99	3.21	11.87	2.87
*LHY*(PH010000383G0320)	73.74	1	199.99	1.44	216.29	1.55	92.41	0.33	56.71	−0.38
*MYB*(PH01000898G0190)	0.01	1	4.39	8.78	6.78	9.41	0.92	6.52	2.36	7.88
*Dof*(PH01000664G0640)	4.53	1	43.09	3.25	46.01	3.35	11.78	1.38	4.19	−0.11
*PIF3*(PH010002918G0050)	1.02	1	10.01	3.30	8.53	3.07	8.32	3.03	1.27	0.32
*MADS14*(PH01000222G1190)	2.07	1	62.02	4.90	59.37	4.84	49.99	4.59	40.27	4.28
*MADS15*(PH01001188G0490	1.52	1	649.59	8.74	877.87	9.17	477.92	8.29	530.17	8.45

## Conclusions

**Figure 4:**
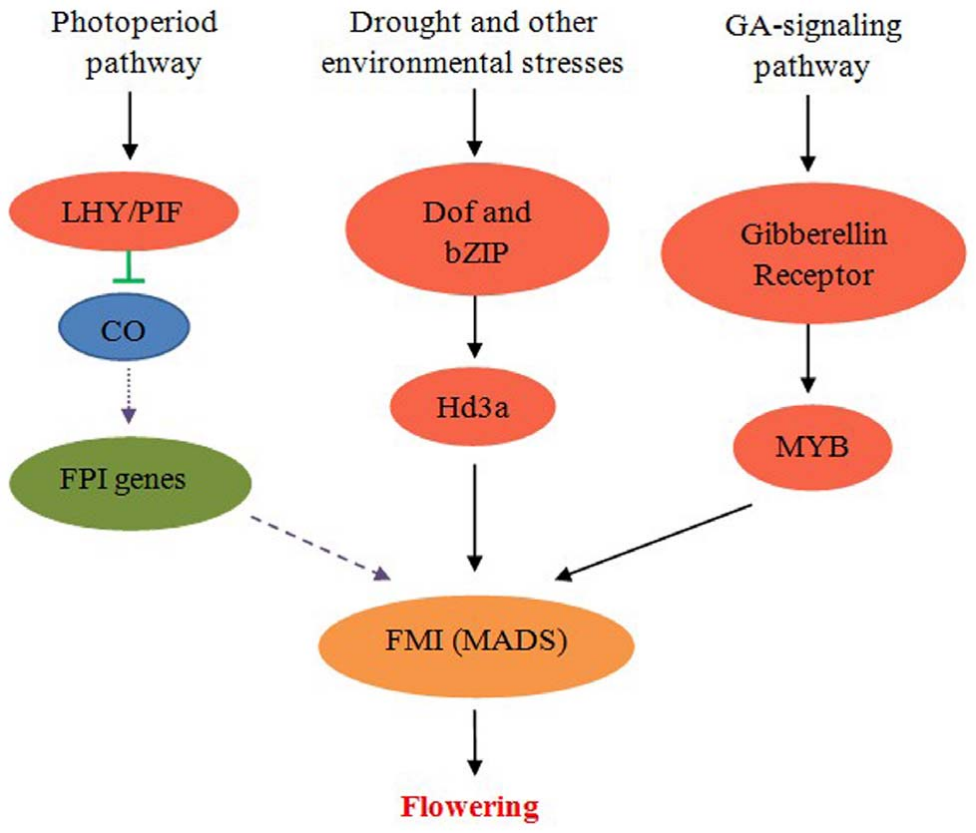
A hypothesized pathways in regulation of flowering in Moso bamboo. Yellow and red oval indicate that the involved genes are more highly expressed in the floral tissues, whereas blue oval indicate that the genes are down-expressed and green oval indicate that the genes are not activated. Purple dashed arrows represent pathways that were not used during flowering. Black arrow represents promotion. Green bar represents suppression.

Little genetic or genomic research has been performed into the fascinating mystery of bamboo flowering despite the economic importance of several bamboo species (as [20]). We investigated the sequences and transcript abundance levels at different developmental stages of Moso bamboo flowers using the Illumina GAII platform. We showed the feasibility of using combination of transcriptome sequencing and RNA-Seq analysis to study the genes involved in floral development in Moso bamboo. More than 714 floral development-related bamboo genes were highly expressed in the panicle tissues. Low expression of CO and FPI genes suggested that FMI was more independent of these known promotion pathways in Moso bamboo flowering. Moreover, high expression of genes involved in stress-responsive pathways and GA-signaling pathway indicated the potential connection between adversity stress, GA and bamboo flowering (shown in Figure 4). The dataset will provide an important resource foundation for future genetic or genomes studies on bamboo species, and will help to give better insight into the mechanism of the flower development of Moso bamboo.

## Materials and Methods

### Ethics Statement

All plant protocols were reviewed and approved by the International Center for Bamboo and Rattan and Key Laboratory of Bamboo and Rattan Science and Technology, State Forestry Administration. All necessary permits were obtained for the field studies from Guangxi Provincial Academy of Forestry and Guilin Forestry Bureau, Dajing County in Guangxi Provence. The field work conducted for sampling did not affect the local ecology and did not involve endangered or protected species.

### Sample preparation and RNA extraction

**Figure 5:**
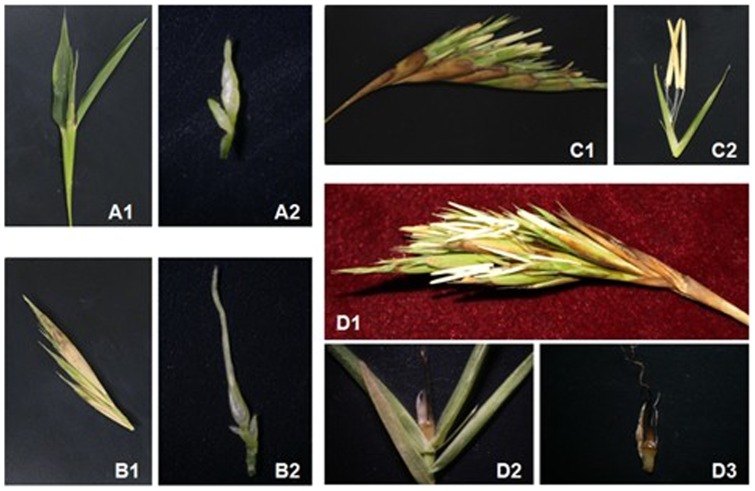
Examples of sampled floral tissues. A1-A2: Representatives of flowers collected to produce the sample for F1 (floral bud formation stage). B1-B2: Representatives of flowers collected to produce the sample for F2 (inflorescence growing stage). C1-C2: Representatives of flowers collected to produce the sample for F3 (bloom stage, flowers with both pistils and stamens emerging from glumes). D1-D3: Representatives of flowers collected to produce the sample for F4 (embryo formation stage).

Moso bamboo samples of non-flowering plants and flowering plants at different flowering developmental stages (floral bud formation, inflorescence development, anthesis and embryo formation stages) were collected in Dajing County, Guilin (E 110°17′-110°47′; N 25°04′-25°48′) in Guangxi Zhuang Autonomous Region from April to August, 2012 (see Figure 5 for details). Four developmental stages were defined, based on the anatomical structure of floral organs: F1, F2, F3, and F4. At the first stage (F1), when the floral bud had begun to form, the plant transits from the vegetative to the reproductive stage. At the second stage (F2), the inflorescence axis continued to extend, lateral buds started to differentiate, and panicles grow from the plant but without flowering. At the third stage (F3), anthesis, flowers with both pistils and stamens emerged from glumes; flowering spikelets were collected at this stage. At the last stage (F4), pistils and stamens had withered, and the embryo was forming.

Samples were immediately frozen in liquid nitrogen and stored at −80°C until further analysis. Total RNA was extracted using the Trizol reagent (Invitrogen, USA). The quality and purified RNA was initially assessed on an agarose gel and NanoDrop 8000 spectrophotometer (NanoDrop, Thermo Scientific, Germany), and then the integrity of RNA samples was further evaluated using an Agilent 2100 Bioanalyzer (Agilent Technologies, USA).

### Library preparation for transcriptome analysis

Samples of two different growth periods were prepared for transcriptome analysis using the Illumina's kit (Illumina, San Diego, CA, USA) according to the manufacturer's instructions. Briefly, mRNA was extracted using magnetic beads with Oligo (dT) (for eukaryotes) or by removing rRNAs from the total RNA (for prokaryotes). Purified mRNA was then mixed with the fragmentation buffer and fragmented into short fragments, which were used as templates for cDNA synthesis. Purified short cDNA fragments were resolved in EB buffer for end reparation and single nucleotide A (adenine) addition, and then they were connected with adapters. Subsequently, suitable cDNA fragments were selected for the PCR amplification as templates. During the QC steps, quantification and qualification of the sample library were assessed using an Agilent 2100 Bioanaylzer and a ABI StepOnePlus Real-Time PCR System. Finally, the library was sequenced using Illumina HiSeq 2000 at Beijing Genomics Institute (BGI) in Shenzhen, China.

### Sequence data quality control and filtering

As Moso bamboo genomic resources were available as the reference, the original image data was transferred into sequence data as raw data or raw reads via base calling after the libraries had been sequenced. Then, the clean reads were selected via analysis and assessment of base composition and quality, and filtering of raw reads by using Illumina PIPILINE software. Since the algorithms used in transcriptome construction of the reads provided by the Illumina platform may be severely inhibited by sequencing errors, a stringent cDNA sequence filtering process was employed to select clean reads. Firstly, reads with adaptors were removed. Secondly, reads in which unknown bases comprised more than 5% of the total were removed. Finally, low quality reads in which the percentage of low quality bases (base quality≤20) was over 30%, were removed. All the clean reads were deposited in the National Center for Biotechnology Information (NCBI) and can be accessed in the Short Read Archive (SRA) linking to BioProject accession number: SRR1187864, SRR1185317.

### Mapping short reads to the Moso bamboo genome and reference gene

The Moso bamboo genome and reference gene set were downloaded from the National Center of Genome Research (http://202.127.18.221/bamboo/index.php), and NCBI site (http://www.ncbi.nlm.nih.gov/nuccore/FO203/436, http://www.ncbi.nlm.nih.gov/nuccore/FO203437, http://www.ncbi.nlm.nih.gov/nuccore/FO203443, http://www.ncbi.nlm.nih.gov/nuccore/FO203444, http://www.ncbi.nlm.nih.gov/nuccore/FO203447, http://www.ncbi.nlm.nih.gov/nuccore/FO203448, http://www.ncbi.nlm.nih.gov/nuccore/FO203439).

After reads containing sequencing adapters and low-quality reads (reads containing Ns>5) were removed, the remaining reads were aligned to the Moso bamboo genome using SOAPaligner/SOAP2 [77], allowing up to two mismatches in each segment alignment. For annotation, transcripts were aligned to four public databases [no redundant protein database (Nr) in NCBI, non-redundant nucleotide database (Nt) in NCBI, Swiss-Prot and Kyoto Encyclopedia of Genes and Genomes (KEGG) pathway database] by BLAST (E-value <10-5). Gene ontology (GO) classification was analyzed by the Blast2GO software (v2.5.0) based on Nr annotation.

### DGEs library preparation and sequencing

Tag library preparation for samples of four different flowering developmental periods (floral bud formation, inflorescence development, anthesis and embryo formation stages) was performed in parallel using the Illumina gene expression sample preparation kit. Briefly, mRNA was extracted using magnetic oligo (dT) beads from total RNA. Purified mRNA was then mixed with the fragmentation buffer and fragmented into short fragments, which were used as templates for cDNA synthesis. Purified short cDNA fragments were resolved in EB buffer for end reparation and single nucleotide A (adenine) addition, and then they were connected with adapters. Subsequently, suitable cDNA fragments were selected for the PCR amplification as templates. During the QC steps, quantification and qualification of the sample library were assessed using an Agilent 2100 Bioanaylzer and an ABI StepOnePlus Real-Time PCR System. Finally, the library was sequenced using Illumina HiSeq 2000 at Beijing Genomics Institute (BGI) in Shenzhen, China.

### Evaluation of DGEs libraries

A statistical analysis of the frequency of each tag in different cDNA libraries was performed to compare the gene-expression in different developmental stages. Statistical comparison was conducted using the method previously described by Audic and Claverie [76]. After the expressional abundances in each library were normalized to transcript per million (RPKM), the most differentially regulated genes (differentially expressed genes, DEGs) were selected. The significance of differential transcript abundance was determined using the FDR (False Discovery Rate) control method [78] to justify the p-values, and only genes with an absolute fold change of ≥2 and a FDR significance score of ≤0.001 were included in subsequent analysis. In addition, p value between two samples was determined using the following formula:
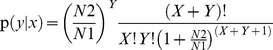



Where N1 and N2 represent the total number of reads mapped to genome in each sample, and x and y represent the number of reads mapped to a common gene in phase 1 and phase 2, respectively.

### Clustering of candidate gene expression profiles

Hierarchical clustering of log-transformed expression data was carried out using the Cluster 3.0 and Treeview programs [79]. Correlations between gene clusters were determined using Pearson's correlation. Heat maps were constructed using the University of Toronto BAR Heatmapper tool (http://www.bar.utoronto.ca/ntools/cgi-bin/ntools_heatmapper.cgi).

### Quantitative real-time PCR (qRT-PCR)

To evaluate the validity of Illumina analysis and assess the expression profiles in terms of specific mRNA abundances, 8 putative genes were selected and detected by qRT-PCR, and to further investigate the expression profiles of these genes, qRT-PCR of flowering tissues at different flowering developmental stages were performed separately using leaves of no-flowering plants as the control. Reverse transcription reactions were performed using 2 mg of RNA by M-MLVRT (Promega, USA) according to the manufacturer's instructions. Sequences of 8 selected genes were obtained from the Moso bamboo genome database (http://www.ncgr.ac.cn/bamboo). Primers, picked by using the Primer 3 software (http://www.genome.wi. mit.edu/cgi-bin/primer/primer3.cgi), as shown in Additional file 13. Tonoplast intrinsic protein (*TIP41*), cited from Fan et al. [80], were used as the internal housekeeping gene control. Real-time PCR reactions were carried out with LightCycler480 System (Roche, USA) using SYBR Premix EX Taq kit (Roche, USA).The 20 µl reaction mixture contained 0.4 µl (10 µM) of each primer and 2 µl (50 ng) cDNA and 10 µl SYBR Green I Master according to the manufacturer's instructions. Amplification reactions were performed as the following: 95°C for 10 s, 60°C for 10 s, and 72°C for 20 s. All reactions were performed in triplicate, both technical and biological. Data was analyzed using Roche manager software.

## Additional Information

### Accession code

The Illumina HiSeq 2000 sequencing data for Moso bamboo flower transeriptome and RNA-seq have been deposited in NIH Short Read Archive database under the accession number SRR1187864 and SRR1185317.

## Supporting Information

Figure S1 Distribution of gene's coverage identification and randomness assessment.
(DOCX)

Figure S2 Overview of Moso bamboo flower RNA-seq sequencing.
(DOCX)

Table S1 GO classification of Moso bamboo genes.
(XLSX)

Table S2 KEGG pathway annotation of Moso bamboo genes.
(XLSX)

Table S3 A list of genes putatively related to flower development in Moso bamboo.
(XLSX)

Table S4 A list of genes showing different transcript abundance in bamboo flowers.
(XLSX)

Table S5 Differentially expressed genes between CK and F1.
(XLSX)

Table S6 Differentially expressed genes between CK and F2.
(XLSX)

Table S7 Differentially expressed genes between CK and F3.
(XLSX)

Table S8 Differentially expressed genes between CK and F4.
(XLSX)

Table S9 List of genes found to be orphan sequences – no homologues in the NCBI database.
(XLSX)

Table S10 List of genes involved in bamboo flowering expression profiles at four different flowering developmental stages.
(XLSX)

Table S11 The names and sequences of primers used in qRT-PCR analysis.
(XLSX)

## References

[1] Maria DL, Artem SK, Dmitriy VV, Tagir HS, Mikhail SG, et al.. (2011) De novo sequencing and characterization of floral transcriptome in two species of buckwheat (Fagopyrum). BMC Genomics 12: 30, 1471–2164. 10.1186/1471-2164-12-30 PMC3027159 21232141

[2] Schuster SC (2008) Next-generation sequencing transforms today's biology. Nat Methods 5(1): 16–18 p. 10.1038/nmeth1156 18165802

[3] Ansorge WJ (2009) Next-generation DNA sequencing techniques. N Biotechnol 25(4): 195–203. 19429539 10.1016/j.nbt.2008.12.009

[4] Metzker ML (2010) Sequencing technologies-the next generation. Nat Rev Genet 11: 31–46. 19997069 10.1038/nrg2626

[5] Rosenkranz R, Borodina T, Lehrach H, Himmelbauer H (2008) Characterizing the mouse ES cell transcriptome with Illumina sequencing. Genomics 92: 187–194. 18602984 10.1016/j.ygeno.2008.05.011

[6] Hegedus Z, Zakrzewska A, Agoston VC, Ordas A, Racz P, et al (2009) Deep sequencing of the zebrafish transcriptome response to mycobacterium infection. Mol Immunol 46: 2918–2930. 19631987 10.1016/j.molimm.2009.07.002

[7] Xiang LX, He D, Dong WR, Zhang YW, Shao JZ (2010) Deep sequencing-based transcriptome profiling analysis of bacteria-challenged lateolabrax japonicus reveals insight into the immune-relevant genes in marine fish. BMC Genomics 11: 472. 20707909 10.1186/1471-2164-11-472 PMC3091668

[8] Tang Q, Ma XJ, Mo CM, Wilson IW, Song C, et al (2011) An efficient approach to finding Siraitia grosvenorii triterpene biosynthetic genes by RNA-seq and digital gene expression analysis. BMC Genomics12: 343. 10.1186/1471-2164-12-343 PMC3161973 21729270

[9] Science Press (2006) Flora of China. Bei Jing: Science Press. Phyllostachys Volume 22, 163–180p.

[10] Lobovikov M, Paudel S, Piazza M, Ren H, Wu J (2007) World Bamboo Resources: A Thematic Study Prepared in the Framework of the Global Forest Resources Assessment 2005. Rome: Food and Agriculture Organization of the United Nations. 1–73p.

[11] International Network for Bamboo and Rattan (INBAR) (1999) Socio-economic Issues and Constraints in the Bamboo and Rattan Sectors: International Network for Bamboo and Rattan's Assessment. No. 23. Bei Jing: International Network for Bamboo and Rattan (INBAR).

[12] Judziewicz E, Clark L, Londono X, Stern M (1999) American Bamboos. Washington, DC: Smithsonian Institution Press.

[13] Lin XC, Chow TY, Chen HH, Liu CC, Chou SJ, et al (2010) Understanding bamboo flowering based on large-scale analysis of expressed sequence tags. Genet Mol Res 9(2): 1085–1093. 20568053 10.4238/vol9-2gmr804

[14] Tian B, Chen Y, Li D, Yan Y (2006) Cloning and characterization of a bamboo Leafy Hull Sterile1 homologous gene. DNA Sequence 17: 143–151. 17076257 10.1080/10425170600699877

[15] Tian B, Chen Y, Yan Y, Li D (2005) Isolation and ectopic expression of a bamboo MADS-box gene. Chin Sci Bull 50: 145–151.

[16] Lin EP, Peng HZ, Jin QY, Deng MJ, Li T, et al (2009) Identification and characterization of two bamboo (Phyllostachys praecox) AP1/SQUA-like MADSbox genes during floral transition. Planta 231(1): 109–120. 19855996 10.1007/s00425-009-1033-0

[17] Lin XC, Chow TY, Chen HH, Liu CC, Chou SJ, et al (2010) Understanding bamboo flowering based on large-scale analysis of expressed sequence tags. Genet Mol Res 9(2): 1085–1093. 20568053 10.4238/vol9-2gmr804

[18] Xu H, Chen LJ, Qu LJ, Gu HY, Li DZ (2010) Functional conservation of the plant EMBRYONIC FLOWER2 gene between bamboo and Arabidopsis. Biotechnol Lett 32(12): 1961–1968. 20676919 10.1007/s10529-010-0362-1

[19] Nadgauda RS, Parasharami VA, Mascarenhas AF (1990) Precocious flowering and seeding behaviour in tissue-cultured bamboos. Nature 344: 335–336.

[20] Zhang XM, Zhao L, Larson-Rabin Z, Li DZ, Guo ZH (2012) De novo sequencing and characterization of the floral transcriptome of Dendrocalamus latiflorus (Poaceae: Bambusoideae). PLoS One 7(8): e42082. 22916120 10.1371/journal.pone.0042082 PMC3419236

[21] Peng ZH, Lu Y, Li LB, Zhao Q, Feng Q, et al (2013) The draft genome of the fast-growing non-timber forest species Moso bamboo (Phyllostachys heterocycla). Nat Genet 45(5): 456–461. 23435089 10.1038/ng.2569

[22] Gielis J, Goetghebeur P, Debergh P (1999) Physiological aspects and experimental reversion of flowering in Fargesia murieliae (Poaceae, Bambusoideae). Syst Geogr Plants 68: 147–158.

[23] Franklin DC (2004) Synchrony and asynchrony: Observations and hypotheses for the flowering wave in a long-lived semelparous bamboo. J Biogeogr 31: 773–786.

[24] Kanehisa M, Goto S, Kawashima S, Okuno Y, Hattori M (2004) The KEGG resource for deciphering the genome. Nucleic Acids Res 32: D277–280. 14681412 10.1093/nar/gkh063 PMC308797

[25] Zhang DF, Li B, Jia GQ, Zhang TF, Dai JR, et al (2008) Isolation and characterization of genes encoding GRF transcription factors and GIF transcriptional coactivators in Maize (Zea mays L.). Plant Sci 175(6): 809–817.

[26] Theiβen G (2001) Development of floral organ identity, stories from the MADS house. Curr Opin Plant Biol 4(1): 75–85. 11163172 10.1016/s1369-5266(00)00139-4

[27] Parenicová L, de Folter S, Kieffer M, Horner DS, Favalli C, et al (2003) Molecular and phylogenetic analyses of the complete MADS-box transcription factor family in Arabidopsis: new openings to the MADS world. Plant Cell 15(7): 1538–1551. 12837945 10.1105/tpc.011544 PMC165399

[28] Beth AK, Jennifer CF (2005) Molecular Mechanisms of Flower Development: An Armchair Guide. Nature 6: 688–698. 10.1038/nrg1675 16151374

[29] Pelucchi N, Fornara F, Favalli C, Masiero S, Lago C, et al (2002) Comparative analysis of rice MADS-box genes expressed during flower development. Sex Plant Reprod 15: 113–122.

[30] Fornara F, Parenicova L, Falasca G, Pelucchi N, Masiero S, et al (2004) Functional characterization of OsMADS18, a member of AP1/SQUA subfamily of MADS box genes. Plant Physiol. 135: 2207–2219. 10.1104/pp.104.045039 PMC520791 15299121

[31] Dreni L, Jacchia S, Fornara F, Fornari M, Ouwerkerk PB, et al (2007) The D-lineage MADS-box gene OsMADS13 controls ovule identity in rice. Plant J 52: 690–699. 17877710 10.1111/j.1365-313X.2007.03272.x

[32] Yanagisawa S (1995) A novel DNA-binding domain that may form a single zinc finger motif. Nucleic Acids Res 23(17): 3403–3410. 7567449 10.1093/nar/23.17.3403 PMC307217

[33] Yanagisawa S (2002) The Dof family of plant transcription tactors. Trends Plant Sci 7(12): 555–560. 12475498 10.1016/s1360-1385(02)02362-2

[34] Ahmad M, Rim Y, Chen H, Kim JY (2013) Functional characterization of Arabidopsis Dof transcription factor AtDof4.1. Russian Journal of Plant Physiology 60(1): 116–123.

[35] Riechmann JL, Heard J, Martin G, Reuber L, Jiang C, et al (2000) Arabidopsis transcription factors: genome-wide comparative analysis among eukaryotes. Science 290: 2105–2110. 11118137 10.1126/science.290.5499.2105

[36] Gardiner J, Sherr I, Scarpella E (2010) Expression of Dof genes identifies early stages of vascular development in Arabidopsis leaves. Int J Dev Biol 54: 1389–1396. 20563990 10.1387/ijdb.093006jg

[37] Papi M, Sabatini S, Altamura MM, Hennig L, Schafer E, et al (2002) Inactivation of the phloem-specific Dof zinc finger gene DAG1 affects response to light and integrity of the testa of Arabidopsis seeds. Plant Physiol 128: 411–417. 11842145 10.1104/pp.010488 PMC148904

[38] Yanagisawa S, Sheen J (1998) Involvement of maize Dof zinc finger proteins in tissue-specific and light-regulated gene expression. Plant Cell 10(1): 75–89. 9477573 10.1105/tpc.10.1.75 PMC143930

[39] Park DH, Lim PO, Kim JS, Cho DS, Hong SH, et al (2003) The Arabidopsis COG1 gene encodes a Dof domain transcription factor and negatively regulates phytochrome signaling. Plant J 34: 161–171. 12694592 10.1046/j.1365-313x.2003.01710.x

[40] Skirycz A, Jozefczuk S, Stobiecki M, Muth D, Zanor MI, et al (2007) Transcription factor AtDOF4; 2 affects phenylpropanoid metabolism in Arabidopsis thaliana. New Phytol 175(3): 425–438. 17635218 10.1111/j.1469-8137.2007.02129.x

[41] Imaizumi T, Schultz TF, Harmon FG, Ho LA, Kay SA (2005) FKF1 F-Box protein mediates cyclic degradation of a repressor of CONSTANS in Arabidopsis. Science 309: 293–297. 16002617 10.1126/science.1110586

[42] Fornara F, Panigrahi KC, Gissot L, Sauerbrunn N, Ruhl M, et al (2009) Arabidopsis DOF transcription factors act redundantly to reduce CONSTANS expression and are essential for a photoperiodic flowering response. Dev Cell 17: 75–86. 19619493 10.1016/j.devcel.2009.06.015

[43] Yang J, Yang MF, Wen PZ, Fan C, Shen SH (2011) A putative flowering-time-related Dof transcription factor gene, JcDof3, is controlled by the circadian clock in Jatropha curcas. Plant Sci 181(6): 667–674. 21958709 10.1016/j.plantsci.2011.05.003

[44] Miao Y, Laun T, Zimmermann P, Zentgraf U (2004) Targets of the WRKY53 transcription factor and its role during leaf senescence in Arabidopsis. Plant Mol Biol 55: 853–867. 15604721 10.1007/s11103-004-2142-6

[45] Robatzek S, Somssich IE (2001) A new member of the Arabidopsis WRKY transcription factor family, AtWRKY6, is associated with both senescence and defence related processes. Plant J 28: 123–133. 11722756 10.1046/j.1365-313x.2001.01131.x

[46] Eulgem T, Rushton PJ, Robatzek S, Somssich IE (2000) The WRKY superfamily of plant transcription factors. Trends Plant Sci 5: 199–206. 10785665 10.1016/s1360-1385(00)01600-9

[47] Deslandes L, Olivier J, Theulieres F, Hirsch J, Feng DX, et al (2002) Resistance to ralstonia solanacearum in Arabidopsis thaliana is conferred by the recessive RRS1-R gene, a member of a novel family of resistance genes. Proc Natl Acad Sci 99: 2404–2409. 11842188 10.1073/pnas.032485099 PMC122377

[48] Miao Y, Zentgraf U (2007) The antagonist function of Arabidopsis WRKY53 and ESR/ESP in Leaf Senescence Is Modulated by the Jasmonic and Salicylic Acid Equilibrium. Plant Cell 19(3): 819–830. 17369373 10.1105/tpc.106.042705 PMC1867371

[49] Eulgem T, Rushton PJ, Schmelzer E, Hahlbrock K, Somssich IE (1999) Early nuclear events in plant defense signalling: Rapid gene activation by WRKY transcription factors. EMBO J 18: 4689–4699. 10469648 10.1093/emboj/18.17.4689 PMC1171542

[50] Maleck K, Levine A, Eulgem T, Morgan A, Juerg S, et al (2000) The transcriptome of Arabidopsis thaliana during systemic acquired resistance. Nat Genet 26: 403–409. 11101835 10.1038/82521

[51] Robatzek S, Somssich IE (2002) Targets of AtWRKY6 regulation during plant senescence and pathogen defense. Genes Dev 16: 1139–1149. 12000796 10.1101/gad.222702 PMC186251

[52] Cui XW, Zhang Y, Qi FY, Gao J, Chen YW, et al (2013) Overexpression of a moso bamboo (Phyllostachys edulis) transcription factor gene PheWRKY1 enhances disease resistance in transgenic Arabidopsis thaliana. Botany 91(7): 486–494.

[53] Ulker B, Somssich IE (2004) WRKY transcription factors: from DNA binding towards biological function. Curr Opin Plant Biol 7: 491–498. 15337090 10.1016/j.pbi.2004.07.012

[54] Yang PZ, Chen CH, Wang ZP, Fan BF, Chen ZX (1999) A pathogen- and salicylic acid-induced WRKY DNA-binding activity recognizes the elicitor response element of tobacco class I chitinase gene promoter. Plant J 18(2): 141–149.

[55] Rouster J, Leah R, Mundy J, Cameron-Mills V (1997) Identification of a methyl-jasmonate-responsive region in the promoter of a lipoxygenase-1 gene expressed in barley grain. Plant J 11: 513–523. 9107039 10.1046/j.1365-313x.1997.11030513.x

[56] Rushton PJ, Macdonald H, Huttly AK, Lazarus CM, Hooley R (1995) Members of a new family of DNA-binding proteins bind to a conserved ciselement in the promoters of a-Amy2 genes. Plant Mol Biol 29: 691–702. 8541496 10.1007/BF00041160

[57] Li DJ, Yang CH, Li XB, Gan Q, Zhao XF, et al (2009) Functional characterization of rice OsDof12. Planta 229: 1159–1169. 19198875 10.1007/s00425-009-0893-7

[58] Zhang W, Sun Y, Timofejeva L, Chen C, Grossniklaus U, et al (2006) Regulation of Arabidopsis tapetum development and function by DYSFUNCTIONAL TAPETUM1 (DYT1) encoding a putative bHLH transcription factor. Development 133(16): 3085–3095. 16831835 10.1242/dev.02463

[59] Peng J (2009) Gibberellin and jasmonate crosstalk during stamen development. J Integr Plant Biol 51: 1064–1070. 20021553 10.1111/j.1744-7909.2009.00881.x

[60] Bender J, FINK GR (1998) ATR1 activates typtophan gene expression in Arabidopsis. Proc Natl Acad Sci 95: 5565–5660. 10.1073/pnas.95.10.5655 PMC20434 9576939

[61] Borevitz JO, Xia Y, Blount J, Dixon RA, Lamb C (2000) Activation tagging identifies a conserved MYB regulator of phenylpropanoid biosynthesis. Plant Cell 12: 2383–2393. 11148285 10.1105/tpc.12.12.2383 PMC102225

[62] Simpson GG, Dean C (2002) Arabidopsis, the rosetta stone of flowering time? Science 296: 285–289. 11951029 10.1126/science.296.5566.285

[63] Fornara F, de Montaigu A, Coupland G (2010) SnapShot: Control of flowering in Arabidopsis. Cell 141: 550, 550e1-550e2. 10.1016/j.cell.2010.04.024 20434991

[64] Janzen DH (1976) Why bamboos wait so long to flower. Ann Rev Ecol Syst 7: 347–391.

[65] Ehrenreich IM, Hanzawa Y, Chou L, Roe JL, Kover PX, et al (2009) Candidate gene association mapping of Arabidopsis flowering time. Genetics 183: 325–335. 19581446 10.1534/genetics.109.105189 PMC2746157

[66] Mouradov A, Cremer F, Coupland G (2002) Control of flowering time: interacting pathways as a basis for diversity. Plant Cell 14: S111–S130. 12045273 10.1105/tpc.001362 PMC151251

[67] Paul KB, Ruth MB, Joshua SM, Caroline D (2004) Multiple pathways in the decision to flower: enabling, promoting, and resetting. Plant Cell 16: S18–S31. 15037730 10.1105/tpc.015958 PMC2643402

[68] Robson F, Costa MMR, Hepworth SR, Vizir I, Pineiro M, et al (2001) Functional importance of conserved domains in the flowering-time gene CONSTANS demonstrated by analysis of mutant alleles and transgenic plants. Plant J 28(6): 619–631. 11851908 10.1046/j.1365-313x.2001.01163.x

[69] Samach A, Onouchi H, Gold SE, Ditta GS, Schwarz-Sommer Z, et al (2000) Distinct roles of CONSTANS target genes in reproductive development of Arabidopsis. Science 288: 1613–1616. 10834834 10.1126/science.288.5471.1613

[70] Richards DE, King KE, Ait-ali T, Harberd NP (2001) How gibberellin regulates plant growth and development: a molecular genetic analysis of gibberellin signaling. Annu Rev Plant Physiol Plant Mol Biol. 52: 67–88. 10.1146/annurev.arplant.52.1.67 11337392

[71] Tsuji H, Aya K, Ueguchi-Tanaka M, Shimada Y, Nakazono M, et al (2006) MGAMYB controls different sets of genes and is differentially regulated by microRNA in aleurone cells and anthers. Plant J 47(3): 427–444. 16792694 10.1111/j.1365-313X.2006.02795.x

[72] Alexandrov NN, Brover VV, Freidin S, Troukhan ME, Tatarinova TV, et al (2009) Insights into corn genes derived from large-scale cDNA sequencing. Plant Mol Biol 69: 179–194. 18937034 10.1007/s11103-008-9415-4 PMC2709227

[73] Bowman JL, Alvarez J, Weigel D, Meyerowitz EM, Smyth DR (1993) Control of flower development in Arabidopsis thaliana by APETALA1 and interacting genes. Development 119: 721–743.

[74] Jeon JS, Jang S, Lee S, Nam J, Kim C, et al (2000) Leafy hull sterilel is a homeotic mutation in a rice MADS box gene affecting rice flower development. Plant Cell 12(6): 871–884. 10852934 10.1105/tpc.12.6.871 PMC149090

[75] Dreni L, Pilatone A, Yun D, Erreni S, Pajoro A, et al (2011) Functional analysis of all AGAMOUS subfamily members in rice reveals their roles in reproductive organ identity determination and meristem determinacy. Plant Cell 23(8): 2850–2863. 21810995 10.1105/tpc.111.087007 PMC3180796

[76] Audic S, Claverie JM (1997) The significance of digital gene expression profiles. Genome Res 7(10): 986–995. 9331369 10.1101/gr.7.10.986

[77] Li R, Yu C, Li Y, Lam TW, Yiu SM, et al (2009) SOAP2: An improved ultrafast tool for short read alignment. Bioinformatics 25(15): 1966–1967. 19497933 10.1093/bioinformatics/btp336

[78] Benjamini Y, Yekutieli D (2001) The control of the false discovery rate in multiple testing under dependency. Ann Statist 29: 1165–1188.

[79] Eisen MB, Spellman PT, Brown PO, Botstein D (1998) Cluster analysis and display of genome-wide expression patterns. Proc Natl Acad Sci USA 95(25): 14863–14868. 9843981 10.1073/pnas.95.25.14863 PMC24541

[80] Fan CJ, Ma JM, Guo QR, Li XT, Wang H, et al (2013) Selection of Reference Genes for Quantitative Real-Time PCR in Bamboo (*Phyllostachys edulis*). PLoS One 8(2): e56573. 23437174 10.1371/journal.pone.0056573 PMC3577859

