# Supplementary figures and images for: Characterization of the Floral Transcriptome of Moso Bamboo (Phyllostachys edulis) at Different Flowering Developmental Stages by Transcriptome Sequencing and RNA-Seq Analysis
Source: PLoS One. 2014 Jun 10;9(6):e98910. doi: 10.1371/journal.pone.0098910 (PMC4051636; doi:10.1371/journal.pone.0098910)

Gene coverage


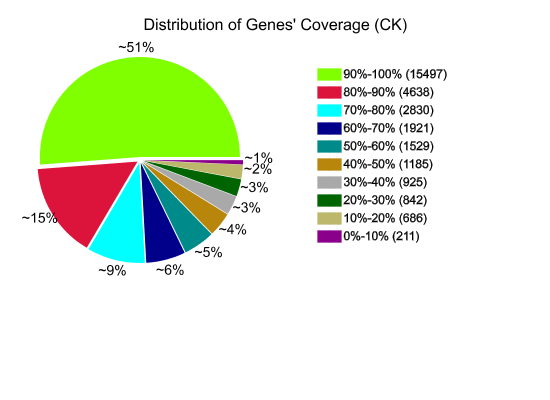

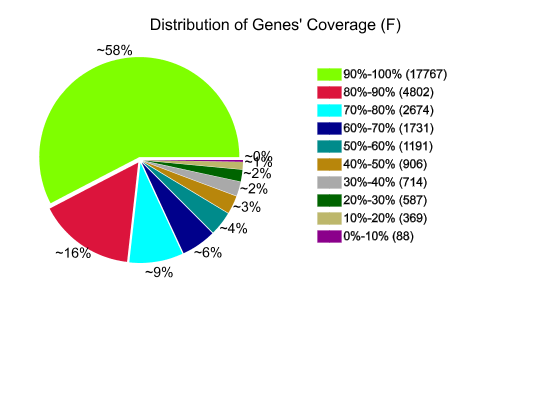


Randomness assessment


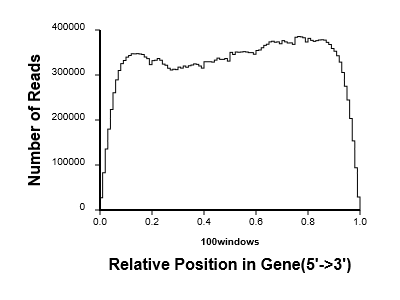

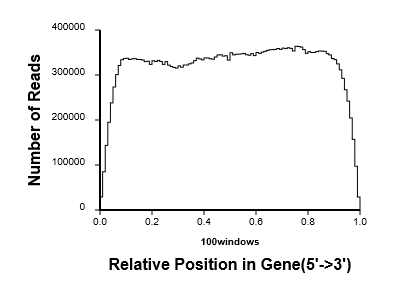


F

CK

Supplement: Figure S1 — Distribution of gene's coverage identification and randomness assessment. (DOCX) [file pone.0098910.s001.docx]
