# Supplementary material for: Characterization of the Floral Transcriptome of Moso Bamboo (Phyllostachys edulis) at Different Flowering Developmental Stages by Transcriptome Sequencing and RNA-Seq Analysis
Source: PLoS One. 2014 Jun 10;9(6):e98910. doi: 10.1371/journal.pone.0098910 (PMC4051636; doi:10.1371/journal.pone.0098910)

The quality assessment of reads:


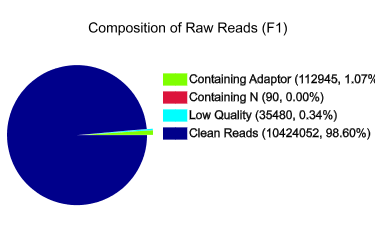

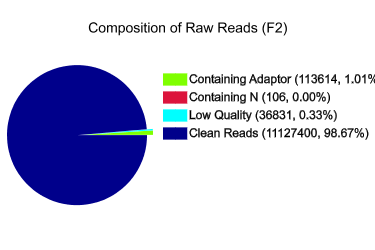

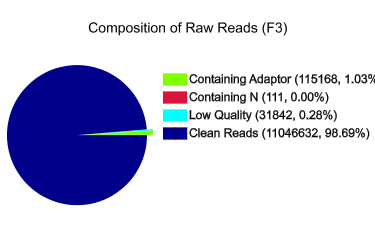

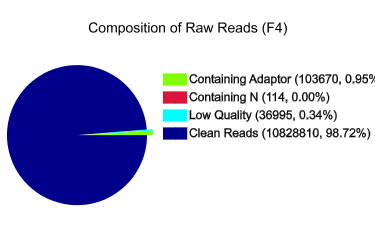


Sequencing saturation analysis


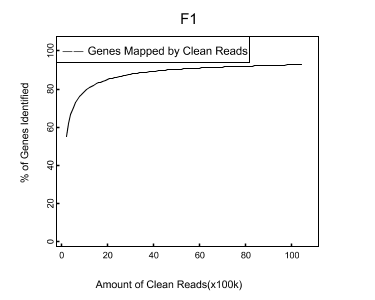

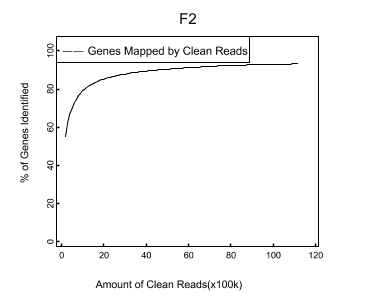

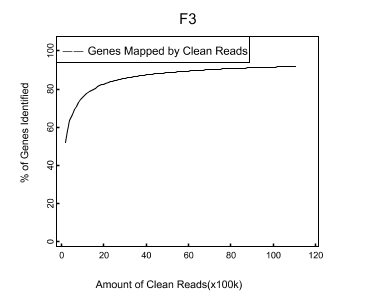

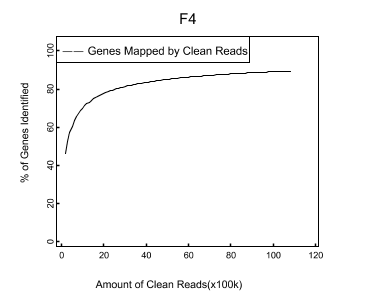


Randomness assessment


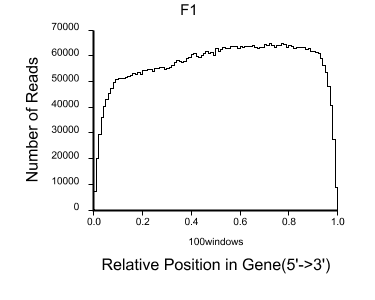

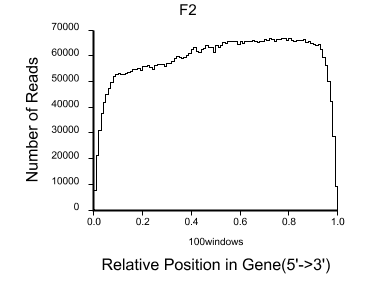

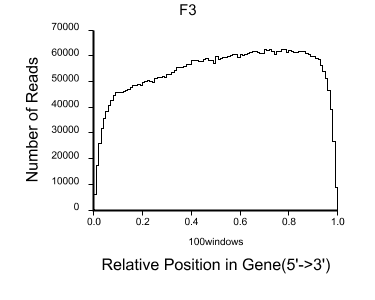

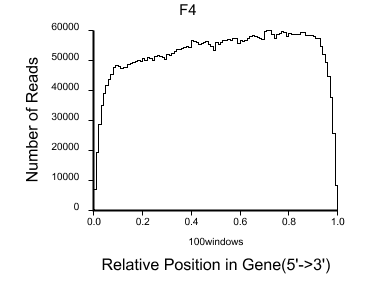


Gene coverage


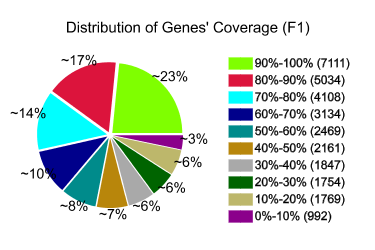

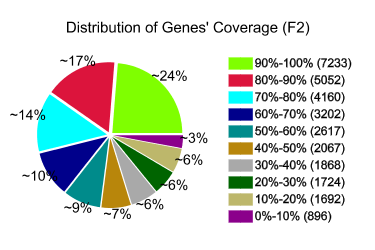

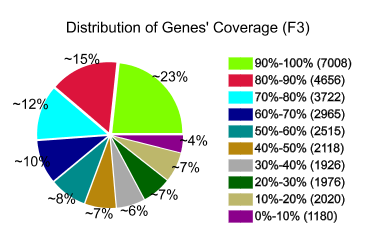

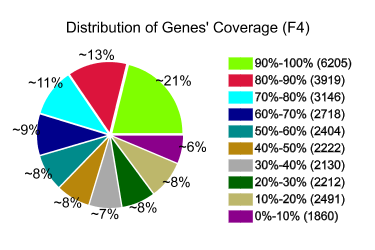

Supplement: Figure S2 — Overview of Moso bamboo flower RNA-seq sequencing. (DOCX) [file pone.0098910.s002.docx]
